# Analysis of the Nutrients and Food Products Intake of Polish Males with Ulcerative Colitis in Remission

**DOI:** 10.3390/nu11102333

**Published:** 2019-10-01

**Authors:** Dominika Głąbska, Dominika Guzek, Gustaw Lech

**Affiliations:** 1 Chair of Dietetics, Department of Dietetics, Faculty of Human Nutrition and Consumer Sciences, Warsaw University of Life Sciences (WULS-SGGW), 159c Nowoursynowska Str., 02-776 Warsaw, Poland; 2 Chair of Consumption Research, Department of Organization and Consumption Economics, Faculty of Human Nutrition and Consumer Sciences, Warsaw University of Life Sciences (WULS-SGGW), 159c Nowoursynowska Str., 02-776 Warsaw, Poland; dominika_guzek@sggw.pl; 3 Department of General, Gastroenterological and Oncological Surgery, Medical University of Warsaw, 1a Banacha Str., 02-097 Warsaw, Poland; gustaw.lech@wum.edu.pl

**Keywords:** ulcerative colitis, colitis ulcerosa, inflammatory bowel disease, diet, nutrients, intake, food products

## Abstract

In spite of the lack of evidence of diet therapy efficacy to sustain remission of ulcerative colitis (UC), the dietary counseling may be beneficial, as a number of patients restrict intake of some products with no medical consultation. The aim of the present study was to analyze the nutrients and food products intake of Polish males with UC in remission in comparison with a control group. The UC group of 44 male patients with the confirmed remission, as well as the pair-matched group of 44 male controls, matched by their age and concurrent diseases, was recruited for the study. Their dietary intake was assessed based on three-day dietary records (to analyze the nutrients and food products intake) and information about food products excluded from their diet, and compared between respondents. It was observed that the intake of food products did not differ between the compared groups, except for the intake of potatoes and sugar, which was higher (*p* = 0.0033, *p* = 0.0092, respectively) in UC patients (median of 209 g and 11 g, respectively) than the control males (median of 100 g and 1 g, respectively). However, it did not influence differences of energy value and nutrients intake between groups, except for the intake of lactose and vitamin B_2_ per 1000 kcal, which was lower (*p* = 0.0425, *p* = 0.0444, respectively) in UC patients (median of 1.8 g and 0.7 g/1000 kcal) than the control males (median of 3.6 g and 0.8 g/1000 kcal). It was observed that the differences in food products intake between the UC individuals in remission and healthy controls were only minor and did not contribute to any significant differences in their nutrients intake. It was concluded that UC patients should be educated not only about the potential influence of food products on their well-being but also about healthy diet recommendations.

## 1. Introduction

Ulcerative colitis (UC) and Crohn’s disease (CD) are inflammatory bowel diseases (IBD), which are characterized by alternating periods of exacerbations and remissions that are hard to predict [1]. The role of diet in the etiology and treatment of IBD has been commonly indicated [2], but the evidence-based clinical practice guidelines emphasize that there is no evidence proving the efficacy of diet therapy in sustaining remission in the case of UC [3]. Therefore, it is stated that patients with UC should not follow any restrictive diet [4], but a number of patients voluntarily restrict the intake of some products [3]. This causes an improperly balanced diet following [5], which consequently [6] leads to deficiencies of calcium, iron, magnesium, zinc, folic acid, and vitamins B_12_, A, and D [7]. At the same time, the disease itself also causes micronutrient deficiencies, dependent on the course of disease and its complications [8].

The IBD patients may choose to restrict their diet in an attempt to control symptoms, which disturb their everyday lives, including work, education, and social relationships [9]. Moreover, recent studies have indicated that about 50% of patients with IBD believe that changing dietary habits is more important than taking medications [10] and diet triggers exacerbation [11].

The existing dietary recommendations for IBD patients in remission are very limited [12] and seem to be in agreement with the general dietary recommendations for healthy individuals [13]. It is commonly believed that a western diet increases the incidence of IBD [14]. Consequently, a number of nutritional factors associated with meat and meat products are indicated as harmful for IBD patients [15], and hence plant-based diets are advised [16]. In addition, alcoholic beverages, as well as food additives, have been shown to have an influence on IBD [17]. It is stated that a high intake of vegetables, fruits, and other products that are sources of soluble fiber may be beneficial to IBD patients, while low fermentable oligosaccharides, disaccharides, monosaccharides and polyols (FODMAP) diet may also be considered [4]. Some authors have also reported other diets, such as specific carbohydrate diet (SCD) [18], as helpful, but there is no sufficient evidence to recommend them.

In spite of the previously indicated lack of evidence of the effectiveness of diet, it has been stated that dietary counseling has an association with symptomatic improvement observed in UC patients. However, this improvement does not result from any single nutrient or food product included to, or excluded from the diet [19], but may emerge from the influence of a number of nutritional factors [20] or may be caused by the improved emotional well-being of patients after application of the holistic approach [21]. Taking this into account, habitual dietary changes are needed to support medical therapy, as it may allow patients to have their diet at least properly balanced based on general dietary recommendations [22]. It is indicated that both dietitians and physicians should guide their patients to follow a properly balanced diet, in order to improve the UC therapy, if possible, or just to not worsen their general condition and quality of life [23]. However, they should know which food products or nutrients should be focused on to recommend a properly balanced diet and provide adequate counseling according to the needs of their patients. Such knowledge is necessary especially for formulating the personalized dietary recommendations that should be based on analysis of current nutritional behaviors, but also preferences, barriers, and objectives [24]. So, dietitians and physicians should previously understand the baseline dietary habits, so that they may be able to provide adequate dietary recommendations and deliver intervention, which would motivate and enable the patient for adequate changes of his eating pattern. 

The aim of the present study was to analyze the nutrients and food products intake of Polish males with UC in remission in comparison with a control group. It was hypothesized that there are specific differences between males with UC in remission and healthy males that may be basis for the future nutritional counseling in this group.

## 2. Materials and Methods 

### 2.1. Study Design

The presented study was conducted at the Dietetic Outpatient Clinic of the Department of Dietetics, Faculty of Human Nutrition and Consumer Sciences, Warsaw University of Life Sciences (WULS-SGGW). The study was conducted in accordance with guidelines of the Declaration of Helsinki, while all the procedures were approved by the Ethical Commission of the Central Clinical Hospital of the Ministry of Interior in Warsaw (No. 35/2009) and the Ethical Commission of the National Food and Nutrition Institute in Warsaw (No. 1604/2009). All participants provided their written informed consent to participate in the study.

### 2.2. Study Participants

The study was conducted in the group of 44 male UC patients, as it was stated that for them such analysis may be especially interesting as male IBD patients rarely undertake any dietetic modifications or other non-pharmacological treatment options [25]. Participants were recruited after being invited by their gastroenterologists, in the procedure of a network convenience sampling, in three Warsaw Gastroenterology Outpatient Clinics: (1) Gastroenterology Outpatient Clinic of the Maria Skłodowska-Curie Memorial Cancer Center and Institute of Oncology in Warsaw, (2) Gastroenterology Outpatient Clinic of the Central Clinical Hospital of the Ministry of Interior and Administration in Warsaw, and (3) Gastroenterology Outpatient Clinic of the Public Central Teaching Hospital in Warsaw. At the same time, a pair-matched control group was recruited in four Warsaw general medical centers.

The individuals included in the control group were assessed using similar inclusion and exclusion criteria as participants in the UC group [26], but with the aim of ensuring that they did not have IBD, and were pair-matched with the UC group. Each control participant was matched with a UC patient with respect to age and concurrent diseases. For this procedure, the concurrent diseases were clustered into following groups: diseases of the blood and blood-forming organs (D50–D89, based on International Statistical Classification of Diseases and Related Health Problems (ICD-10) [27]), disorders of thyroid gland (E00–E07), diabetes mellitus and other disorders of glucose regulation and pancreatic internal secretion (E10–E16), disorders of lipoprotein metabolism and other lipidemias (E78), mental and behavioral disorders (F00–F99), diseases of the nervous system (G00–G99), hypertensive diseases (I10–I15), diseases of the respiratory system (J00–J99), diseases of the digestive system other than noninfective enteritis and colitis, as well as other diseases of the intestines (K00–K46; K65–K93), diseases of the skin and subcutaneous tissue (L00–L99), diseases of the musculoskeletal system and connective tissue (M00–M99), inflammatory diseases of female pelvic organs, as well as disorders of the genitourinary system (N99). Afterwards, each patient was pair-matched with the control subject with the concurrent diseases attributed to the same groups. However, for each UC patient, from the pair-matching procedure those diseases were excluded, which were defined by their gastroenterologists as a complication of UC. The diseases were indicated for each participant separately and the same disease may have been excluded from the procedure for one patient (as for him it was a complication of UC), but not excluded for the other patient (as for him it was an independent disease). Moreover, body mass index (BMI) was not included for matching as a factor that may also be influenced by UC. After the pair-matching procedure, a total of 44 control participants were included in the study.

The first stage of the study was the assessment of BMI and body composition, as well as the comparison of the obtained results between the UC patients and control individuals. As it was found that the BMI, as well as the share of individuals with excessive body mass, did not differ between groups, while only minor differences were found in the body composition assessed using bioelectrical impedance method (extracellular water and body cell mass index) [26], it was assumed that the groups were comparable and that their dietary intake may also be compared.

**Figure 1:**
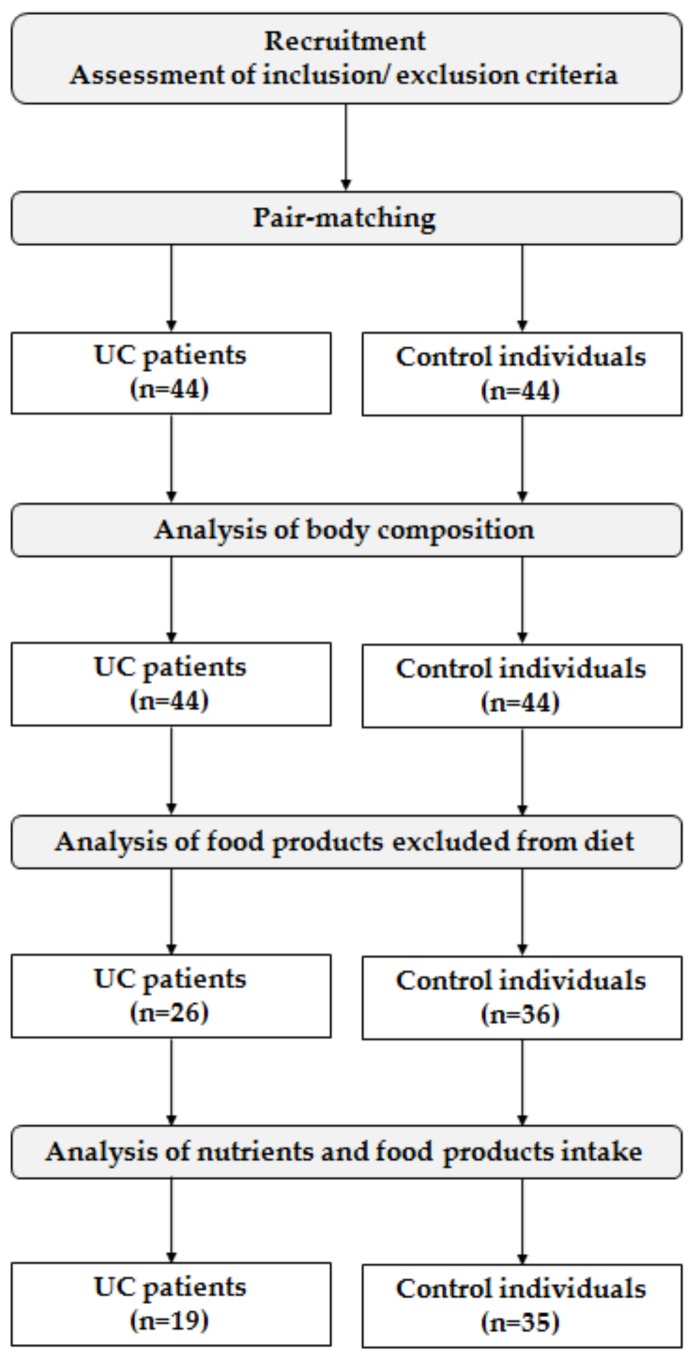
Study design and number of participants.

The second stage of the study was the assessment of the respondents’ diet, based on their three-day dietary records (collected to analyze the nutrients and food products intake) and information about food products excluded from the diet, and comparison between the groups, as presented in Figure 1.

### 2.3. Diet Analysis

To assess the diet, the participants were asked to conduct their three-day dietary records (self-reported data). They received detailed instruction in a standard structured form and were asked to record all the food products and beverages consumed during three random typical days that are not consecutive (two weekdays and one weekend day) [28]. They were asked not to change their typical dietary habits for the purpose of recording, as well as to scrupulously report all the food products with the description of dishes and thermal treatment applied.

The study participants were also asked to record the serving sizes of all the food products consumed, as well as the food products consumed as elements of dishes—they were allowed to declare the serving sizes either in grams (if they had a kitchen scale to verify, or if they had consumed packed products, with the information about weight provided by the manufacturer) or using a typical household measure. After obtaining the three-day dietary records, the serving sizes declared by the participants were verified by a professional dietitian, who used the Polish Atlas of Food Products and Dishes Portion Size and attributed the mass in grams [29].

The energy value of the three-day dietary records, as well as the intake of nutrients, was calculated as the mean daily value. The Polish dietician software Dieta 5.0 (National Food and Nutrition Institute, Warsaw, Poland) with the Polish database of the nutritional value of food products [30] was used for analysis. The following nutrients were included to the analysis: total protein, share of energy from protein, animal protein, plant protein, total fat, share of energy from fat, saturated fatty acids (SFA), monounsaturated fatty acids (MUFA), polyunsaturated fatty acids (PUFA), cholesterol, total carbohydrates, share of energy from carbohydrates, sucrose, lactose, starch, fiber, alcohol, share of energy from alcohol, sodium, potassium, calcium, phosphorus, magnesium, iron, zinc, copper, manganese, vitamin A, E, D, B_1_, B_2_, niacin, B_6_, folate, B_12_, and C. The intake of nutrients was recalculated per 1000 kcal of diet to allow comparisons between groups, independent of the energy value of diets [31]. 

At the same time, the unadjusted intake values were also compared as the dietary reference intake values, being independent from the energy value of diet, present the required total dietary supply [32]. They were also compared with the Polish age-dependent reference intake values [33], while either the estimated average requirement (EAR), or adequate intake (AI) levels were used to compare.

The three-day dietary records were also used to calculate the intake of food products, while the following products were included to the analysis: milk and dairy beverages, cottage cheese, rennet cheese, eggs, meat, processed meat products, fish and fish products, vegetables, legumes, fruits, potatoes, bread, other cereal products, oil, margarine, butter, cream, sugar, jam and honey, chocolate sweets, cakes and cookies, tea, coffee, alcoholic beverages, sweetened beverages, nuts, and mushrooms. Afterwards, the intake of food products was recalculated per 1000 kcal of diet to allow comparisons between groups, independent of the energy value of diets, similarly as done for the intake of nutrients.

Additional information about food products deliberately excluded from the diet was provided by all the respondents. The participants received a structured questionnaire and were asked to indicate the specific food products which were excluded by them from diet or the intake of which was limited due to gastroenterological symptoms (open-ended question). Then, these food products were attributed to a food product group to verify if within the group any food products were excluded from the diet.

### 2.4. Statistical Analysis

The normality of distribution of the obtained data was verified by using the Shapiro-Wilk test. Based on the distribution, either a parametric Student’s t-test, or non-parametric Mann-Whitney U test was applied. The shares of respondents declaring exclusion of products from diet, as well as the shares of respondents characterized by the intake below reference intake values were compared by using chi^2^ test. The *p* ≤ 0.05 was accepted in order to verify the significance. The statistical analysis was conducted while using Statistica software version 8.0 (StatSoft Inc., Tulsa, OK, USA) and Statgraphics Plus for Windows 5.1 (Statgraphics Technologies Inc., The Plains, VA, USA).

## 3. Results

The macronutrients intake in the groups of UC males and control males is presented in Table 1. It was observed that the intake of macronutrients did not differ between the compared groups.

The macronutrients intake per 1000 kcal in the groups of UC males and control males is presented in Table 2. It was observed that the intake of macronutrients did not differ between the compared groups, except for the intake of lactose per 1000 kcal, which was lower (*p* = 0.0425) in UC patients (median of 1.8 g/1000 kcal) than the control males (median of 3.6 g/1000 kcal).

The minerals intake in the groups of UC males and control males is presented in Table 3. It was observed that the intake of minerals did not differ between the compared groups.

The minerals intake per 1000 kcal in the groups of UC males and control males is presented in Table 4. It was observed that the intake of minerals did not differ between the compared groups.

The vitamins intake in the groups of UC males and control males is presented in Table 5. It was observed that the intake of vitamins did not differ between the compared groups.

The vitamins intake per 1000 kcal in the groups of UC males and control males is presented in Table 6. It was observed that the intake of vitamins did not differ between the compared groups, except for the vitamin B_2_ intake per 1000 kcal, which was lower (*p* = 0.0444) in UC patients (median of 0.7 g/1000 kcal) than the control males (median of 0.8 g/1000 kcal).

The food products intake in the groups of UC males and control males is presented in Table 7. It was observed that the intake of food products did not differ between the compared groups, except for the intake of potatoes and sugar, which was higher (*p* = 0.0033, *p* = 0.0092, respectively) in UC patients (median of 209 g and 11 g, respectively) than the control males (median of 100 g and 1 g, respectively).

The food products intake per 1000 kcal in the groups of UC males and control males is presented in Table 8. It was observed that the intake of food products did not differ between the compared groups, except for the intake of potatoes and sugar per 1000 kcal, which was higher (*p* = 0.0047, *p* = 0.0065, respectively) in UC patients (mean of 77.7 ± 43.8 g/1000 kcal, median of 6.3 g/1000 kcal, respectively) than the control males (mean of 45.1 ± 35.7 g/1000 kcal, median of 0.4 g/1000 kcal, respectively).

The food products declared as excluded from diet in a group of UC males and control males are presented in Table 9. It was observed that food products declared as excluded from diet did not differ between compared groups.

The comparisons of the minerals and vitamins intake with the age-dependent reference intake estimated average requirement (EAR)/adequate intake (AI) values [33] in a group of UC males and control males are presented in Table 10. For a number of individuals in both groups the inadequate intake was stated, especially for potassium, calcium, magnesium, and vitamin D. At the same time, the share of participants characterized by inadequate intake did not differ between compared groups.

## 4. Discussion

In the presented study it was observed that the differences in food products intake between the UC patients in remission and healthy controls were only minor and did not contribute to any significant differences in their nutrients intake. The hypothesis that there are specific differences between males with UC in remission and healthy males that may be basis for the future nutritional counseling in this group was not confirmed. 

Lack of differences in macronutrients intake is comparable with the results of the study by Taylor et al. [34], in which they observed no differences in macronutrients intake between CD patients and the representative sample. In the study of Principi et al. [35] on IBD patients while compared with healthy individuals, the protein and carbohydrate intake was similar between the groups, while higher fat intake contributed to the higher energy value of diets. Similarly, in the study of Vahid et al. [36] on UC patients, a higher intake of macronutrients was observed compared to control individuals, which corresponded to the higher energy value of their diet. As observed both in the own study and indicated studies of other authors [34,35,36] the energy value of the diets of UC patients is not lower than for control groups, but it is either comparable (as in the presented own study), or even higher [35,36]. It results in the corresponding observations for BMI, being for UC patients either comparable, or even higher than for control groups, that was stated in a number of studies [26,37,38].

However, while analyzing the studies of other authors, it must be noted that the results observed for intake of vitamins and minerals are not consistent, as some of them state lower intake by IBD individuals than the control groups [34,39,40,41,42], but the lower intake was stated mainly for CD patients [34,40,41,42], whereas for UC patients, even higher intake was reported compared to the control groups [36]. Thus, the lack of differences indicated in the present study may confirm the general lack of a regular undeviating trend of differences between the IBD individuals and controls.

Furthermore, no statistically significant differences were observed between the groups in the present study, with respect to the food products declared as excluded from the diet. Such a lack of differences may be interpreted as that the UC individuals were not following any specific diet or any diet that is not typical for healthy individuals in this age group. Similarly, in the study by Limdi et al. [11], which was conducted on a group of 400 IBD patients, in spite of the fact that 68% of respondents declared avoiding specific products to avoid exacerbations, they indicated that they mainly excluded spicy food products (41%), fatty food products (29%) and alcohol (21%), which are not products specifically advised to be avoided for IBD patients but typical products advised to be excluded from diet for general population. The frequency of avoiding certain products was similar in the study by Zallot et al. [43], but at the same time, they observed a higher frequency of belief of necessity to avoid some vegetables, being even higher than for spicy food products or alcohol, which are in fact the mainly excluded products as stated in the study by Limdi et al. [11]. Additionally, in the present study, no UC respondent declared avoiding vegetables other than legumes while being in remission, which is in accordance with the approach observed for healthy individuals. Therefore, it may be stated that even if the respondents believed that they should avoid vegetables, they did not follow it. Some lack of agreement between the dietary beliefs and the actual diet which is followed may result from the lack of effectiveness of the nutritional behaviors which are believed as beneficial by patients, as in general they declare that they adopt such behaviors which are effective for them [44]. However, other authors also indicated that the decisions to exclude some food products from the diets are based on the nutritional beliefs of the patients [45].

In the research of other authors, it was observed that the most common dietary approach of IBD patients is to neither follow any specific diet nor include any beneficial food products, but just avoid some of them [46]. Similarly, in the present study, the respondents excluded some food products from their diets, and when these products were analyzed within the food product groups, they were not found to differ from the foods excluded by the control group. Such exclusion of specific food products may be interpreted as a proper approach, as patients may have individual tolerance to some food products, and so personalized recommendations should be followed [47]. However, if the patients do not cover their nutritional requirements, avoiding food products is not a proper solution as it may lead to not properly balanced diet following, which is not in accordance with the general nutritional recommendations [5].

It should be emphasized that IBD patients should, above all, not only avoid products that cause gastroenterological symptoms but also provide adequate amounts of nutrients, because nutrient deficiencies may occur even in apparently well-nourished patients or in patients without laboratory abnormalities [48]. Thus, even if malnutrition is not observed [26], and the diet does not differ from that of healthy individuals, the risk of deficiencies is higher [49].

While the food products intake was compared, the differences between UC patients and control males were only minor and were associated with the higher intake of potatoes and sugar in the UC group. Similarly, in the study by Opstelten et al. [50], for some food product groups there were differences observed between IBD patients and control individuals; the IBD patients were characterized by a significantly higher intake of carbonated beverages, which was corresponding to the difference in sugar intake stated in the present study (potatoes were not analyzed as a separate group in the referred study). Such an observation may be attributed to the commonly indicated high intake of simple carbohydrates by IBD patients, which is even attributed to the higher risk of the disease [51,52].

In spite of the fact that between the analyzed groups there was no difference in the intake of milk and dairy beverages, it should be indicated that 32% of UC males and 20% of control males declared exclusion of these food products from their diet. In addition, although lactose intolerance is more common in women than in men [53], the frequency of 20% noted in the control group was lower than the frequency of lactose intolerance in the general Polish population [54] and the frequency of lactose intolerance in the IBD population is known to be higher than in the healthy population [55]. Due to the common limitation of the dairy products intake, that was indicated in the study by Opstelten et al. [50], while compared with healthy individuals, the calcium intake of IBD patients is commonly lower than the recommended level [56], as was also observed in the present study. This reduced calcium intake is one of the contributors to the higher osteoporosis risk in IBD patients than the general population [57]. Moreover, calcium intake is observed to be lower in patients believing that the consumption of lactose-containing food products induces the symptoms of IBD than in those who do not believe so [58]. Thus, the reduced calcium intake is associated with the popularity of such beliefs and the common restriction of dairy products in IBD patients [59]. Furthermore, in the present study, while recalculated per 1000 kcal of diet, the lactose intake was lower for UC individuals than for control ones. At the same time, in the systematic review and meta-analysis by Szilagyi et al. [60], it was stated that lactose maldigestion in IBD patients is dependent on ethnic background rather than the disease and that restrictions of dairy food products may even adversely affect the disease outcome, which also does not support reducing the intake of dairy beverages.

Moreover, as some potential factors that may allow for the induction of remission or its maintenance in IBD patients, such as low refined carbohydrates content [61], are indicated, they should be included in the diet of patients. Based on the results of the present study, it may be concluded that the diet of UC individuals does not differ from the diet of healthy ones, and so the UC patients do not benefit from the possible positive effect of the dietary changes. At the same time, taking into account the fact that diet of IBD patients commonly does not satisfy their nutritional needs, it must be indicated that nutritional counseling is needed for this group.

While comparing the obtained results with the results of other studies, it should be emphasized that the presented own study provided a more complex analysis than a number of other studies, including the study by Principi et al. [35] on 150 IBD patients, as not only macronutrients (protein, fat and carbohydrates), but also number of micronutrients (minerals and vitamins) were here analyzed. It allowed to state that, in spite of the fact that for macronutrients no differences between UC males and control individuals were stated, there were some minor differences for micronutrients (as observed for vitamin B_2_). Moreover, not only unadjusted intake was assessed, but also intake per 1000 kcal that allowed more reliable comparison between groups. At the same time, not only nutrient intake was analyzed, but also the corresponding food product intake. Last, but not least, it must be indicated that in a number of studies of IBD patients, they are compared with healthy subjects while analyzed combined, while there may be some differences between UC and CD patients, as even dietary risk factors may differ for them [62].

In spite of the fact that the presented study provided the novel observations associated with the issues that were for the time being rarely studied—namely micronutrient intake and food product intake in UC males while compared with the control individuals, the further directions of the research should be suggested. While analyzing the food product intake, it would be valuable to assess separately unprocessed and processed fruits and vegetables (as was conducted for meat products in the presented study). Moreover, conducting similar complex analysis also for female UC patients would allow deeper analysis.

## 5. Conclusions

It was observed that the differences in food products intake between the UC individuals in remission and healthy controls were only minor and did not contribute to any significant differences in their nutrients intake. UC patients were characterized by similarly inadequate intake as healthy controls, that was stated especially for potassium, calcium, magnesium, and vitamin D. Therefore, it can be concluded that UC patients should be educated not only about the potential influence of food products on their well-being but also about healthy diet recommendations.

## Figures and Tables

**Table 1:** The macronutrients intake in ulcerative colitis males and control males.

Nutrient—Daily Intake	Ulcerative Colitis Males		Control Males		p **
	Mean ± SD	Median (Range)	Mean ± SD	Median (Range)	
Energy (kcal)	2821.0 ± 632.0	2850.0 (1683.0–3823.0)	2650.0 ± 919.1	2608.3 * (819.2–6629.0)	0.2319
Total protein (g)	111.9 ± 25.5	110.3 (72.3–170.4)	115.7 ± 48.4	104.2 * (52.2–276.0)	0.6377
Total protein (% energy)	16.2 ± 2.5	15.7 (12.4–222.2)	18.2 ± 5.4	16.8 * (12.8–36.9)	0.4468
Animal protein (g)	77.0 ± 21.4	72.1 (44.0–135.0)	82.7 ± 43.6	69.2 * (30.8–211.3)	0.6377
Plant protein (g)	35.0 ± 8.2	33.3 (19.4–50.4)	32.1 ± 10.5	30.5 (14.0–64.9)	0.3095
Total fat (g)	125.0 ± 40.2	120.2 (62.6–221.0)	115.0 ± 57.0	105.3 * (30.9–365.5)	0.2852
Total fat (% energy)	38.8 ± 6.2	39.3 (27.5–51.8)	38.6 ± 7.3	38.4 (23.2–55.6)	0.9230
SFA (g)	42.8 ± 13.8	42.6 (20.3–72.9)	37.9 ± 18.5	35.6 * (8.2–109.3)	0.1631
MUFA (g)	53.2 ± 19.4	54.3 (23.5–106.2)	49.0 ± 25.1	43.9 * (12.6–155.9)	0.2614
PUFA (g)	19.0 ± 7.7	18.4 (8.0–37.2)	18.8 ± 12.1	16.7 * (5.7–76.5)	0.5931
Cholesterol (mg)	486.4 ± 158.9	448.6 (236.0–776.9)	530.1 ± 267.0	460.2 * (153.5–1516.4)	0.8991
Total carbohydrates (g)	333.3 ± 79.6	340.4 (225.3–521.5)	299.5 ± 90.5	311.3 (75.5–560.9)	0.1783
Total carbohydrates (% energy)	44.6 ± 6.0	45.0 (29.7–56.4)	42.5 ± 7.3	43.34 (29.6–58.5)	0.2711
Sucrose (g)	63.6 ± 32.8	54.1 (11.3–135.7)	50.0 ± 29.0	45.5 (7.4–118.1)	0.1219
Lactose (g)	6.1 ± 4.6	5.1 (0.2–14.9)	13.0 ± 17.1	7.6 * (0.5–79.6)	0.1012
Starch (g)	200.2 ± 65.5	188.9 (68.6–312.1)	167.5 ± 69.0	169.5 (11.9–359.3)	0.0971
Fiber (g)	24.8 ± 6.0	23.5 (15.3–37.4)	25.3 ± 8.1	23.8 (11.2–50.9)	0.8365
Alcohol (g)	1.2 ± 3.9	0.0 * (0.0–15.8)	6.8 ± 14.3	0.0 * (0.0–61.6)	0.1448
Alcohol (% energy)	0.3 ± 1.0	0.0 * (0.0–4.1)	1.8 ± 4.0	0.0 * (0.0–17.9)	0.1499

SFA—saturated fatty acids; MUFA—monounsaturated fatty acids; PUFA—polyunsaturated fatty acids; * non-parametric distribution (verified using Shapiro-Wilk test—*p* ≤ 0.05); ** compared using Student’s t-test (for parametric distributions) or Mann-Whitney U test (for non-parametric distributions).

**Table 2:** The macronutrients intake per 1000 kcal in ulcerative colitis males and control males.

Nutrient—Intake per 1000 kcal	Ulcerative Colitis Males		Control Males		p **
	Mean ± SD	Median (Range)	Mean ± SD	Median (Range)	
Total protein (g)	40.1 ± 6.1	38.5 (30.7–54.9)	44.7 ± 13.7	40.6 * (31.6–92.0)	0.6377
Animal protein (g)	27.6 ± 6.0	26.1 (20.1–41.7)	31.9 ± 13.8	26.5 * (12.8–75.0)	0.6638
Plant protein (g)	12.5 ± 2.1	12.5 (9.4–16.9)	12.4 ± 2.5	12.4 (7.7–18.1)	0.8425
Total fat (g)	43.8 ± 7.0	44.1 (30.9–58.6)	42.4 ± 8.1	42.2 (25.3–56.7)	0.5374
SFA (g)	15.0 ± 2.8	15.0 (10.0–20.4)	14.1 ± 3.7	14.1 (6.2–21.3)	0.3391
MUFA (g)	18.6 ± 3.9	17.7 (11.7–28.1)	18.0 ± 3.8	17.9 (10.2–24.9)	0.6061
PUFA (g)	6.6 ± 1.9	6.4 (3.8–11.4)	6.8 ± 2.2	6.7 * (2.7–13.9)	0.6377
Cholesterol (mg)	176.2 ± 58.1	177.5 (95.5–284.6)	209.2 ± 110.4	198.4 * (86.0–694.4)	0.2934
Total carbohydrates (g)	119.1 ± 15.6	120.1 (80.3–152.0)	114.6 ± 18.6	115.6 (83.3–159.0)	0.3736
Sucrose (g)	22.5 ± 10.4	21.5 (5.4–42.8)	19.1 ± 10.3	17.3 * (4.6–46.5)	0.2048
Lactose (g)	2.2 ± 1.7	1.8 (0.1–5.8)	5.0 ± 6.0	3.6 * (0.2–29.3)	0.0425
Starch (g)	70.9 ± 17.1	68.2 (29.5–103.6)	63.2 ± 19.9	62.5 * (7.9–101.1)	0.2048
Fiber (g)	9.0 ± 2.2	8.9 (5.2–12.7)	9.9 ± 2.8	9.6 * (4.9–19.9)	0.3946
Alcohol (g)	0.4 ± 1.4 *	0.0 (0.0–5.8)	2.6 ± 5.8	0.0 * (0.0–25.8)	0.0616

SFA—saturated fatty acids; MUFA—monounsaturated fatty acids; PUFA—polyunsaturated fatty acids; * non-parametric distribution (verified using Shapiro-Wilk test—*p* ≤ 0.05); ** compared using Student’s t-test (for parametric distributions) or Mann-Whitney U test (for non-parametric distributions).

**Table 3:** The minerals intake in ulcerative colitis males and control males.

Nutrient—Daily Intake	Ulcerative Colitis Males		Control males		p **
	Mean ± SD	Median (Range)	Mean ± SD	Median (Range)	
Sodium (mg)	3197.5 ± 1066.2	3141.5 (1121.7–5145.0)	3506.5 ± 1533.4	3307.0 * (1244.2–9773.0)	0.5261
Potassium (mg)	4099.1 ± 691.2	4090.8 (2892.0–5309.0)	411.1 ± 1410.5	3726.1 * (2047.4–8096.6)	0.4577
Calcium (mg)	676.7 ± 258.3	642.2 (339.7–1112.5)	859.1 ± 519.2	705.0 * (306.6–2479.0)	0.4361
Phosphorus (mg)	1663.9 ± 402.2	1599.6 (1044.4–2645.6)	1807.4 ± 778.0	1631.5 * (963.8–4330.8)	1.0000
Magnesium (mg)	394.1 ± 117.5	360.1 * (270.9–716.1)	394.3 ± 138.4	347.8 * (204.7–832.1)	0.9422
Iron (mg)	15.9 ± 3.9	15.1 (9.9–23.1)	16.6 ± 5.6	15.1 * (6.6–32.3)	0.8634
Zinc (mg)	14.7 ± 4.0	13.7 (9.9–24.3)	15.2 ± 5.1	14.1 * (7.7–29.3)	0.9278
Copper (mg)	1.5 ± 0.4	1.5 (1.1–2.5)	1.5 ± 0.5	1.5 * (0.76–3.0)	0.8138
Manganese (mg)	6.8 ± 2.5	6.6 (2.8–11.2)	7.1 ± 3.5	6.3 * (2.0–16.3)	0.8279

* non-parametric distribution (verified using Shapiro-Wilk test—*p* ≤ 0.05); ** compared using Student’s t-test (for parametric distributions) or Mann-Whitney U test (for non-parametric distributions).

**Table 4:** The minerals intake per 1000 kcal in ulcerative colitis males and control males.

Nutrient—Intake per 1000 kcal	Ulcerative Colitis Males		Control Males		p **
	Mean ± SD	Median (Range)	Mean ± SD	Median (Range)	
Sodium (mg)	1144.2 ± 341.6	1092.4 (562.0–2005.9)	1337.6 ± 358.5	1303.5 (525.5–2470.6)	0.0599
Potassium (mg)	1512.5 ± 370.7	1382.7 (844.3–2193.6)	1648.5 ± 684.9	1487.8 * (796.0–4827.5)	0.7719
Calcium (mg)	244.7 ± 94.9	245.3 (111.9–468.1)	335.1 ± 175.5	294.1 * (104.4–911.6)	0.0621
Phosphorus (mg)	598.0 ± 108.9	582.7 (422.5–825.4)	701.3 ± 229.5	639.3 * (441.6–1490.0)	0.1069
Magnesium (mg)	143.8 ± 44.3	139.7 * (83.2–269.2)	155.3 ± 50.0	144.1 * (83.7–358.3)	0.3018
Iron (mg)	5.7 ± 1.2	5.3 (3.9–8.2)	6.6 ± 2.2	6.2 * (3.9–16.0)	0.1686
Zinc (mg)	5.2 ± 0.9	5.0 (3.9–6.6)	5.9 ± 1.6	5.7 * (3.5–12.0)	0.1031
Copper (mg)	0.6 ± 0.2	0.5 (0.4–1.0)	0.6 ± 0.2	0.6 * (0.3–1.3)	0.3191
Manganese (mg)	2.5 ± 1.1	2.1 * (1.3–5.3)	2.7 ± 1.1	2.6 (0.8–5.5)	0.3462

* non-parametric distribution (verified using Shapiro-Wilk test—*p* ≤ 0.05); ** compared using Student’s t-test (for parametric distributions) or Mann-Whitney U test (for non-parametric distributions).

**Table 5:** The vitamins intake in ulcerative colitis males and control males.

Nutrient—Daily Intake	Ulcerative Colitis Males		Control Males		p **
	Mean ± SD	Median (Range)	Mean ± SD	Median (Range)	
Vitamin A (µg RE)	2099.6 ± 1218.2	1949.4 * (830.8–5403.3)	2139.5 ± 2046.9	1524.8 * (381.3–9785.0)	0.2771
Vitamin E (mg a-TE)	17.4 ± 7.0	15.2 (7.1–30.4)	16.9 ± 9.5	15.9 * (4.7–62.7)	0.6638
Vitamin D (µg)	6.2 ± 5.4	4.0 * (2.0–21.2)	7.7 ± 6.5	5.4 * (1.4–28.6)	0.3414
Vitamin B_1_ (mg)	2.0 ± 0.7	1.8 * (1.1–3.7)	2.0 ± 0.8	1.8 * (0.7–4.0)	0.6903
Vitamin B_2_ (mg)	2.0 ± 0.5	2.0 (1.3–3.6)	2.4 ± 1.1	2.0 * (1.2–5.4)	0.6312
Niacin (mg)	26.6 ± 7.0	24.8 (15.0–42.0)	27.6 ± 14.0	24.1 * (8.9–71.7)	0.6184
Vitamin B_6_ (mg)	2.8 ± 0.6	2.7 (1.7–3.79)	2.8 ± 1.1	2.6 * (1.1–6.6)	0.4743
Folate (µg)	404.2 ± 110.2	362.4 * (279.2–705.2)	408.7 ± 131.1	408.5 * (177.9–890.8)	0.8138
Vitamin B_12_ (µg)	7.2 ± 5.0	5.6 * (2.2–18.4)	8.3 ± 6.1	5.7 * (2.7–28.8)	0.3651
Vitamin C (mg)	118.8 ± 50.2	106.6 (48.6–240.7)	136.0 ± 68.0	120.9 * (32.4–329.4)	0.4468

RE—retinol equivalents; a-TE—α-tocopherol equivalents; * non-parametric distribution (verified using Shapiro-Wilk test—*p* ≤ 0.05); ** compared using Student’s t-test (for parametric distributions) or Mann-Whitney U test (for non-parametric distributions).

**Table 6:** The vitamins intake per 1000 kcal in ulcerative colitis males and control males.

Nutrient—Intake per 1000 kcal	Ulcerative Colitis Males		Control Males		p **
	Mean ± SD	Median (Range)	Mean ± SD	Median (Range)	
Vitamin A (µg RE)	750.7 ± 409.1	608.2 * (262.0–1896.1)	855.4 ± 852.1	565.9 * (230.3–4132.6)	0.6770
Vitamin E (mg a-TE)	6.2 ± 2.2	5.4 (3.4–11.6)	6.3 ± 1.8	6.1 (2.0–10.2)	0.7826
Vitamin D (µg)	2.3 ± 2.2	1.6 * (0.9–9.1)	2.8 ± 1.8	2.5 * (0.8–8.4)	0.3556
Vitamin B_1_ (mg)	0.7 ± 0.2	0.7 * (0.5–1.2)	0.8 ± 0.2	0.7 * (0.5–1.6)	0.4913
Vitamin B_2_ (mg)	0.7 ± 0.2	0.7 * (0.4–1.3)	0.9 ± 0.4	0.8 * (0.5–2.4)	0.0444
Niacin (mg)	9.6 ± 2.0	10.1 (6.9–13.5)	10.7 ± 4.9	9.6 * (4.8–29.4)	0.9278
Vitamin B_6_ (mg)	1.0 ± 0.3	1.0 * (0.7–1.7)	1.1 ± 0.4	1.0 * (0.6–2.8)	0.5868
Folate (µg)	147.2 ± 38.8	145.5 (80.1–247.5)	165.8 ± 77.0	144.6 * (91.6–551.4)	0.4687
Vitamin B_12_ (µg)	2.7 ± 2.2	1.7 * (1.0–8.0)	3.1 ± 1.8	2.7 * (1.3–8.4)	0.0759
Vitamin C (mg)	44.2 ± 20.1	40.9 (13.9–88.8)	58.4 ± 47.9	45.9 * (9.5–277.4)	0.2852

RE—retinol equivalents; a-TE—α-tocopherol equivalents; * non-parametric distribution (verified using Shapiro-Wilk test—*p* ≤ 0.05); ** compared using Student’s t-test (for parametric distributions) or Mann-Whitney U test (for non-parametric distributions).

**Table 7:** The food products intake in ulcerative colitis males and control males.

Food Products—Daily Intake	Ulcerative Colitis Males		Control Males		p **
	Mean ± SD	Median (Range)	Mean ± SD	Median (Range)	
Milk and dairy beverages (g)	72.7 ± 80.9	57.0 * (0.0–205.0)	173.9 ± 263.1	110.0 * (0.0–1200.0)	0.1656
Cottage cheese (g)	28.9 ± 55.6	0.0 * (0.0–172.0)	39.0 ± 71.4	13.0 * (0.0–388.0)	0.1631
Rennet cheese (g)	28.1 ± 29.3	17.0 * (0.0–93.0)	26.4 ± 33.8	13.0 * (0.0–127.0)	0.6111
Eggs (g)	40.0 ± 36.8	47.0 * (0.0–136.0)	51.2 ± 61.1	40.0 * (0.0–277.0)	0.8490
Meat (g)	204.2 ± 158.6	181.2 * (0.0–715.0)	168.3 ± 129.4	125.0 * (10.0–506.2)	0.3279
Processed meat products (g)	108.1 ± 83.8	91.0 (0.0–284.0)	105.3 ± 89.6	77.0 * (0.0–343.0)	0.7375
Fish and fish products (g)	50.9 ± 76.6	0.0 * (0.0–267.0)	44.8 ± 48.3	40.0 * (0.0–200.0)	0.7455
Vegetables (g)	261.8 ± 109.4	255.3 (12.9–490.6)	344.2 ± 201.0	288.2 * (40.0–1080.0)	0.1922
Legumes (g)	2.6 ± 7.8	0.0 * (0.0–28.0)	3.1 ± 9.1	0.0 * (0.0–41.0)	0.5577
Fruits (g)	168.2 ± 186.7	89.4 * (0.0–597.6)	152.9 ± 151.9	94.1 * (0.0–547.1)	0.8989
Potatoes (g)	206.7 ± 90.6	209.0 (0.0–330.0)	122.9 ± 106.8	100.0 * (0.0–400.0)	0.0033
Bread (g)	226.1 ± 91.1	220.0 (57.0–427.0)	203.5 ± 105.0	190.0 * (23.0–521.0)	0.2730
Other cereal products (g)	52.4 ± 40.8	41.0 (0.0–143.0)	46.8 ± 37.7	38.0 * (0.0–154.0)	0.5867
Oil (g)	17.7 ± 17.2	11.0 * (0.0–60.0)	20.4 ± 15.9	20.0 * (0.0–67.0)	0.4094
Margarine (g)	6.5 ± 9.4	3.0 * (0.0–36.0)	10.3 ± 31.9	2.0 * (0.0–189.0)	0.9482
Butter (g)	13.8 ± 15.3	9.0 * (0.0–57.0)	12.9 ± 10.5	10.0 * (0.0–39.0)	0.7367
Cream (g)	10.5 ± 11.8	8.0 * (0.0–45.0)	9.1 ± 14.2	7.0 * (0.0–79.0)	0.4396
Sugar (g)	20.0 ± 18.6	11.0 * (0.0–52.0)	10.6 ± 20.6	1.0 * (0.0–77.0)	0.0092
Jam and honey (g)	11.8 ± 21.5	0.0 * (0.0–90.0)	6.5 ± 16.4	0.0 * (0.0–80.0)	0.1517
Chocolate sweets (g)	15.7 ± 20.5	10.0 * (0.0–67.0)	12.9 ± 20.5	0.0 * (0.0–74.0)	0.2764
Cakes and cookies (g)	39.3 ± 52.8	12.0 * (0.0–184.0)	47.5 ± 65.6	8.0 * (0.0–206.0)	0.8944
Tea (g)	710.4 ± 572.1	733.0 * (0.0–2500.0)	619.5 ± 472.2	583.0 * (0.0–1833.0)	0.5614
Coffee (g)	143.4 ± 169.2	83.0 * (0.0–542.0)	128.0 ± 179.1	0.0 * (0.0–800.0)	0.5705
Alcoholic beverages (g)	11.4 ± 39.4	0.0 * (0.0–167.0)	78.2 ± 179.7	0.0 * (0.0–716.0)	0.0569
Sweetened beverages (g)	97.7 ± 119.7	67.0 * (0.0–384.0)	80.9 ± 105.9	42.0 * (0.0–340.0)	0.6972
Nuts (g)	11.2 ± 27.5	0.0 * (0.0–92.0)	3.7 ± 9.6	0.0 * (0.0–46.7)	0.8168
Mushrooms (g)	2.2 ± 8.2	0.0 * (0.0–36.0)	2.2 ± 5.6	0.0 * (0.0–21.1)	0.9589

* non-parametric distribution (verified using Shapiro-Wilk test—*p* ≤ 0.05); ** compared using Student’s t-test (for parametric distributions) or Mann-Whitney U test (for non-parametric distributions).

**Table 8:** The food products intake per 1000 kcal in ulcerative colitis males and control males.

Food Products—Intake per 1000 kcal	Ulcerative Colitis Males		Control Males		p **
	Mean ± SD	Median (Range)	Mean ± SD	Median (Range)	
Milk and dairy beverages (g)	26.8 ± 32.0	19.3 * (0.0–100.7)	72.9 ± 100.8	36.2 * (0.0–441.2)	0.1045
Cottage cheese (g)	9.8 ± 18.3	0.0 * (0.0–60.3)	13.3 ± 17.4	5.0 * (0.0–67.8)	0.1464
Rennet cheese (g)	10.0 ± 10.4	7.7 * (0.0–33.0)	9.5 ± 11.9	5.9 * (0.0–50.9)	0.6847
Eggs (g)	15.1 ± 14.1	15.6 * (0.0–49.8)	22.4 ± 29.3	15.5 * (0.0–146.5)	0.6901
Meat (g)	70.0 ± 48.6	54.6 * (0.0–189.6)	66.7 ± 51.9	50.4 * (4.5–202.9)	0.5868
Processed meat products (g)	38.7 ± 28.2	30.2 (0.0–87.1)	40.0 ± 29.5	30.3 * (0.0–111.5)	0.9422
Fish and fish products (g)	20.7 ± 35.1	0.0 * (0.0–132.9)	17.5 ± 19.2	16.4 * (0.0–68.9)	0.6064
Vegetables (g)	98.8 ± 52.0	100.5 (5.6–210.2)	147.7 ± 124.7	127.5 * (11.1–755.4)	0.0729
Legumes (g)	0.8 ± 2.5	0.0 * (0.0–9.3)	1.1 ± 3.1	0.0 * (0.0–13.5)	0.5479
Fruits (g)	65.6 ± 79.6	31.0 * (0.0–256.5)	68.1 ± 82.5	35.0 * (0.0–374.8)	1.0000
Potatoes (g)	77.7 ± 43.8	74.1 (0.0–191.9)	45.1 ± 35.7	42.8 (0.0–126.1)	0.0047
Bread (g)	79.7 ± 26.2	72.7 (28.5–131.0)	77.2 ± 31.1	72.6 (19.5–132.4)	0.7696
Other cereal products (g)	18.3 ± 12.5	18.4 (0.0–43.2)	18.1 ± 14.5	15.4 * (0.0–64.0)	0.6572
Oil (g)	6.0 ± 5.4	4.0 * (0.0–16.7)	7.6 ± 4.6	8.1 * (0.0–20.2)	0.1373
Margarine (g)	2.5 ± 3.6	0.9 * (0.0–10.8)	2.6 ± 5.1	0.9 * (0.0–28.5)	0.9852
Butter (g)	5.0 ± 5.1	3.7 * (0.0–17.5)	5.0 ± 4.0	4.0 * (0.0–14.9)	0.7030
Cream (g)	3.9 ± 5.4	2.7 * (0.0–21.5)	3.6 ± 5.5	2.8 * (0.0–30.4)	0.8598
Sugar (g)	7.1 ± 6.4	6.3 (0.0–20.5)	3.5 ± 7.1	0.4 * (0.0–28.2)	0.0065
Jam and honey (g)	4.7 ± 9.1	0.0 * (0.0–38.6)	1.8 ± 3.7	0.0 * (0.0–13.9)	0.1488
Chocolate sweets (g)	5.7 ± 7.9	2.8 * (0.0–26.4)	4.9 ± 7.8	0.0 * (0.0–27.7)	0.4567
Cakes and cookies (g)	12.3 ± 15.5	4.3 * (0.0–48.1)	17.7 ± 24.6	5.2 * (0.0–76.6)	0.8053
Tea (g)	272.6 ± 264.2	237.7 * (0.0–1195.3)	234.6 ± 165.4	213.9 (0.0–601.8)	0.9566
Coffee (g)	58.9 ± 71.0	23.8 * (0.0–232.6)	59.5 ± 100.1	0.0 * (0.0–488.3)	0.6039
Alcoholic beverages (g)	3.9 ± 13.4	0.0 * (0.0–56.7)	27.6 ± 64.4	0.0 * (0.0–300.5)	0.0524
Sweetened beverages (g)	33.7 ± 42.4	21.7 * (0.0–124.4)	33.5 ± 45.6	20.5 * (0.0–171.3)	0.9244
Nuts (g)	4.2 ± 10.6	0.0 * (0.0–39.5)	1.6 ± 4.5	0.0 * (0.0–22.7)	0.8168
Mushrooms (g)	0.7 ± 2.6	0.0 * (0.0–11.2)	1.0 ± 2.5	0.0 * (0.0–10.5)	1.0000

* non-parametric distribution (verified using Shapiro-Wilk test—*p* ≤ 0.05); ** compared using Student’s t-test (for parametric distributions) or Mann-Whitney U test (for non-parametric distributions).

**Table 9:** The food products declared as excluded from diet in ulcerative colitis males and control males.

Food Products	Share of Respondents Declaring Exclusion of Products from Diet		p *
	Ulcerative Colitis Males	Control Males	
Milk and dairy beverages	31.6	20.0	0.5371
Cottage cheese	68.4	40.0	0.0873
Rennet cheese	31.6	34.3	1.0000
Eggs	10.5	11.4	1.0000
Meat	5.3	0.0	0.7542
Processed meat products	10.5	2.9	0.5803
Fish and fish products	57.9	40.0	0.3302
Vegetables	0.0	0.0	1.0000
Legumes	89.5	82.9	0.8006
Fruits	10.5	17.1	0.8006
Potatoes	5.3	20.0	0.2916
Bread	0.0	0.0	1.0000
Other cereal products	5.3	2.9	1.0000
Oil	15.8	5.7	0.4665
Margarine	36.8	34.3	1.0000
Butter	26.3	11.4	0.3080
Cream	36.8	37.1	1.0000
Sugar	15.8	42.9	0.0868
Jam and honey	52.6	71.4	0.2788
Chocolate sweets	31.6	54.3	0.1894
Cakes and cookies	42.1	45.7	1.0000
Tea	10.5	14.3	1.0000
Coffee	42.1	51.4	0.7116
Alcoholic beverages	89.5	65.7	0.1147
Sweetened beverages	42.1	45.7	1.0000
Nuts	78.9	80.0	1.0000
Mushrooms	78.9	80.0	1.0000

* compared using chi^2^ test.

**Table 10:** The comparisons of the minerals and vitamins intake with the age-dependent reference intake estimated average requirement (EAR)/adequate intake (AI) values [33] in ulcerative colitis males and control males.

Nutrients		Ulcerative Colitis Males		Control Males		p *
		Intake below Reference Values	Intake above Reference Values	Intake below Reference Values	Intake above Reference Values	
Minerals	Sodium	5.3	94.7	5.7	94.3	1.0000
	Potassium	84.2	15.8	77.1	22.9	0.7933
	Calcium	94.7	5.3	80.0	20.0	0.2916
	Phosphorus	0.0	100.0	0.0	100.0	1.0000
	Magnesium	42.1	57.9	45.7	54.3	1.0000
	Iron	0.0	100.0	0.0	100.0	1.0000
	Zinc	0.0	100.0	0.0	100.0	1.0000
	Copper	0.0	100.0	0.0	100.0	1.0000
Vitamins	Vitamin A	0.0	100.0	2.9	97.1	1.0000
	Vitamin E	10.5	89.5	17.1	82.9	0.8006
	Vitamin D	63.2	36.8	57.1	42.9	0.8890
	Vitamin B_1_	0.0	100.0	8.6	91.4	0.4895
	Vitamin B_2_	0.0	100.0	0.0	100.0	1.0000
	Niacin	0.0	100.0	5.7	94.3	0.7586
	Vitamin B	0.0	100.0	5.7	94.3	0.7586
	Folate	10.5	89.5	20.0	80.0	0.6102
	Vitamin B_12_	0.0	100.0	0.0	100.0	1.0000
	Vitamin C	21.1	78.9	20.0	80.0	1.0000

* compared using chi^2^ test.
